# Planting? or Implantation? Who First Gave This Operation to the Profession?

**Published:** 1898-02

**Authors:** W. H. Robinson

**Affiliations:** 1344 Park Street, Alameda, Cal.


### Planting? or Implantation? Who First Gave This Operation to the Profession?

The following is part of an article as published in the *Dental Jairus*, Cal., August, 1880:

"THE DENTAL JAIRUS—A CRITICISM" (page) 339, BY W. H. ROBINSON, D.D.S."

"The almost universal success of these operations," (transplanting and replanting) "is astonishing, when we look at the records of them, and see that not only the way they are performed is careless, but in many cases in utter violation of every principle that should guide us in such operations. When the genius of the profession is directed to these operations, so as to give them the attention they merit, we will soon know the modes and principles so well that transplanting or replanting will be operations with fewer failures than now follow ordinary nerve devitalization. Furthermore, we will soon see teeth transplanted into alveoli, that have been without teeth for years. Let me make this point emphatic, Mr. A. has lost an incisor, and wears a cumbrous plate. He presents himself to the dentist, and says he wants a real tooth there, instead of that cumbrous plate-tooth-arrangement. The dentist says call at such a time, and I will perform the operation. In the mean time, the dentist has found a suitable tooth for transplanting into Mr. A's mouth. He calls; the dentist makes a socket in the alveolus, inserts the tooth, and if a real live, useful tooth, is a desirable success, then transplanting is a success, for it gives that result. So confident am I that teeth can be successfully planted into an alveolus where the teeth have been out for years, in a socket prepared by the dentist to receive them, that I would have no fears or hesitation in performing it, whenever proper opportunity occured."

" Implanting is Dr. Younger's term, first performed by him in 1885. I used the term "Planting" to describe the same operation in 1880, and I orally described this operation before the California State Dental Association in 1881 (Dr. Younger being present), in the discussion of Dr. Thomas' mode of using dry teeth, an imperfect report of which is on page 131, minutes of California State Dental Association, 1881.

W. H. Robinson, D. D. S.

1344 Park Street, Alameda, Cal., July, 1897.

